# Fear of COVID-19 and Its Relationship with eHealth Literacy: Impact on Mental Health and the Role of Personal Infection Experience

**DOI:** 10.1192/j.eurpsy.2025.1279

**Published:** 2025-08-26

**Authors:** M. Theodoratou, K. Deligianni, K. Flora, K. Megari, G. Tsitsas

**Affiliations:** 1 Social Sciences, Hellenic Open University, Patras, Greece; 2 Psychology, Neapolis University of Pafos, Pafos, Cyprus; 3 Hellenic Open University, Patras; 4 Psychology, University of West Macedonia, Florina; 5 Psychology, Aristotelian University of Thessaloniki, Thessaloniki; 6 Psychology, Harokopeion University, Athens, Greece

## Abstract

**Introduction:**

The advent of the Coronavirus Disease 2019 (Covid-19) pandemic in late 2019 precipitated a profound transformation in the daily lives of individuals across the world, with notable implications for global mental health. The fear of the virus became a significant source of anxiety and stress, compounded by the rapid dissemination of information, both accurate and inaccurate, through digital media. The growing reliance on digital platforms for health information during the pandemic underscored the significance of eHealth literacy, which pertains to the capacity to locate, comprehend, and utilize health data from digital sources. As reliance on digital platforms for health information increased, it became imperative to examine the potential impact of digital health literacy on fear and anxiety levels related to the novel coronavirus disease (2019-nCoV), also known as Coronavirus Disease 2019 (Covid-19).

**Objectives:**

The objective of this study is to examine the relationship between fear of the Coronavirus disease 2019 (Covid-19) pandemic and mental health, while investigating the influence of eHealth literacy on these fears.

**Methods:**

The study was conducted on a sample of 158 individuals from the general population, representing a diverse range of demographic characteristics. The data were collected using a structured questionnaire comprising three sections: demographic information, the Fear of Coronavirus Scale (FCV-19S), and the eHealth Literacy Scale (eHeals). A statistical analysis was conducted to investigate the relationships between demographic factors, levels of fear, and eHealth literacy. Additionally, a comparative analysis was performed to assess the differences between individuals who had and had not contracted the virus.

**Results:**

The analysis revealed no statistically significant differences in the levels of fear regarding the novel coronavirus disease (Covid-19) or eHealth literacy between different demographic groups, including gender, age, education, and health status. Nevertheless, a statistically significant discrepancy was observed in the level of fear between individuals who had contracted the virus and those who had not. Specifically, those who had not been infected exhibited higher levels of fear compared to those who had previously contracted the virus. No significant differences were identified in eHealth literacy based on infection status.

**Conclusions:**

In conclusion, the findings of this study indicate that while demographic factors do not appear to influence the level of fear or digital health literacy, personal experience with a confirmed case of infection does. Those who have not contracted the virus tend to experience greater fear, suggesting that familiarity with the disease may reduce anxiety. These findings underscore the necessity for interventions.

**Disclosure of Interest:**

None Declared

